# Reports of Odd Cases

**Published:** 1898-06

**Authors:** E. L. Quitman

**Affiliations:** Professor of Materia Medica, Therapeutics, and Toxicology, Chicago Veterinary College, Chicago, Ill.


### REPORTS OF ODD CASES.

By E. L. Quitman, M.D.C.,
PROFESSOR OF MATERIA MEDICA, THERAPEUTICS, AND TOXICOLOGY, CHICAGO VETERINARY COLLEGE, CHICAGO, ILL.

A MERE synopsis of the following cases can only be given, as specific data were not kept. But as they are all unique, I trust they will be interesting:

CASE I. occurred about five years ago; a bay mare, between twelve and fourteen years of age, was first noticed by her owner (a physician) to be very sensitive about her neck; flies seemed to irritate her much more than usual, and she would shrink and show considerable pain upon the skin of the neck being stroked with the hand, which he attributed to the irritation of flies. Upon noticing this he protected the neck with a fly-sheet, but in spite of this precaution the mare grew worse, so much so that when I was called in the slightest touch upon the neck would cause rapid and involuntary raising and lowering of the *hind* leg of the opposite side, and a slight or persistent pressure would cause the animal to fall in a tetanic spasm, lasting from a minute to about five minutes. After the convulsions she would lie motionless for about two minutes, then regain her feet. At the time I was called in her pupils remained dilated, thus interfering with her sight, and she was more nervous than normally; appetite good.

*Diagnosis.* Hyperæsthesia, due to some central nervous trouble.

*Treatment.* Put her on gelsemium and bromide of potash internally, and locally used a wash containing hydrocyanic acid, which had to be applied with an atomizer, as she could not stand the slightest touch without falling in convulsions.

This treatment was persisted in for about two weeks, the doses being increased until they were enormous; but to no avail. After this length of time I prescribed the following:

| | |
|---|---|
| ℞.—Iod. resub. . . . . . . . . | ʒiij. |
| Potas. iod. . . . . . . . . | ℥jss. |
| Aq. . . . . . . . . . . | ℥xij.—M. |

Sig.—One ounce three times daily in four or five ounces of water on an empty stomach.

Five days after the commencement of this treatment there was marked improvement, and at the end of two weeks from its commencement the mare was discharged as cured. She was then put back to her regular work, continuing in this for about three months (until about October), when she again started to show the symptoms already described.

The owner not caring to bother with her any more, at my request allowed me to remove her to the Chicago Veterinary College, where she was kept for some time and used as a clinical subject.

She was kept there, as near as I remember, for about six weeks, when she was destroyed.

*Postmortem* revealed all visceral organs in a normal condition, the generative organs being very carefully examined, as it was suggested that possibly the ovaries might be the cause of the trouble; they, however, were perfectly normal.

The lesions found were as follows: Great effusion into the *cervical* meningeal spaces due, of course, to a chronic meningitis of that region, and in the atlo-axoid space there was found a stellate fibro-granular tumor (if it may be so termed) that seemed to blend with the dura mater.

This tumor, to the eye, seemed to consist of a coarse fibrous meshwork with a granular or sandy, lumpy material interspersed in the meshes.

Thus it can be seen from its location it was the primary cause of the trouble—*i. e.*, from pressure upon the meninges and interference with the circulation it set up a subacute and thence chronic meningitis.

The brain showed very slight effusion into the central ventricle.

CASE II. *Hemorrhage from the lungs that persisted for about thirty-six hours, terminating in death.*—This was a large, phlegmatic draught-horse, about fifteen years old; had always been very healthy. Administered various drugs, such as ergot, gallic acid, lead acetate, etc., even injecting a small quantity of diluted Monsell's solution into the trachea.

The interesting feature of this case was that the blood lacked the fibrin-forming ferments, *and did not clot* (hæmatolysis). I kept some of it in a bucket for several days, using different means to make it clot, but it remained in a fluid state.

CASE III.—A bay draught-horse, seven or eight years old, weighing about 1500 pounds, came in one evening with slight superficial bleeding from the sheath. The driver upon being asked regarding its occurrence, stated that the horse had made a spread-eagle slip, but got up and went along all right.

I thought but little of the case and prescribed an astringent antiseptic wash, expecting to hear no more of it. Was called the next morning and found the sheath enormously swollen, so much so that it could hardly have been contained in a bushel-basket, and with but little abnormal heat; naturally the animal urinated in the sheath.

There being but little abnormal heat, and the parts not being painful, I excluded septic and inflammatory conditions and made a diagnosis of rupture of bloodvessels. I treated the parts locally with various measures—hot bathing, cold bathing, astringent applications, etc., and iodine and iodide of potassium internally for about two or three weeks, when out of desperation I made up my mind to use the knife. I made large incision into the sheath, finding it very œdematous. Not being satisfied with this, and finding the penis greatly enlarged and having a peculiar feel, I made an incision deep into it, from which a dark-colored serum started to ooze; thence upon pressure a clot came. I now enlarged the opening enough to admit my whole hand, and was amazed at what I found. I removed three-fourths of a water-bucketful of blood-clot and serum, finding the urethra completely macerated away from its surrounding tissues, from the end of the penis to the pubis; after all clots were removed I flushed the parts thoroughly with an antiseptic solution and continued antiseptic treatment. After a reasonable time, much quicker than expected, the animal was returned to his work.

CASE IV.—A bay gelding, eight years old, had been for about seven or eight months in a very emaciated condition ; appetite good ; teeth in first-class condition.

I had prescribed tonics and diets of all kinds for him; treatment had been persisted in for a considerable length of time, but without improving him in the least. Pulse a little weaker and faster than normal, which I attributed to his debility. No elevation of temperature. Had practically given up the case, when one evening I was called in a hurry to the horse in question ; found him with a bad case of flatulent colic ; ordered him brought out of the box-stall ; when upon reaching the floor he suddenly stopped, braced himself, showed great anxiety, and with great force vomited about two quarts of matter from his stomach. Upon examination I found the vomited matter to be about one-half *pus* and *blood*.

Inasmuch as his pulse immediately ran down, I made an unfavorable prognosis, but prescribed appropriate remedies, and was surprised to have him recover, and, more, he began almost immediately to gain flesh, and in the short time of three or four weeks was in excellent flesh, even fat.

His trouble was undoubtedly due to a submucous or intermuscular abscess of the stomach.

CASE V.—Was hurriedly called to a mule, but did not arrive until about two hours after he had taken sick.

*History*. I was told that the mule first showed considerable nervousness and anxiety, but *was not bloated* ; finally he commenced to vomit, which continued for about three-quarters of an hour at short intervals.

I found the mule free from pain and flatulence, deathly cold ; pulse almost imperceptible ; mucous membranes blanched, and temperature two degrees subnormal (taken with a Hicks certified thermometer), and the animal trembling violently.

I naturally considered it a rupture of the stomach and a hopeless case ; but was asked to prescribe, which I did, as follows, on general principles. Gave a hypodermic injection of spirits of glonoin ʒij (1 per cent.) and then

℞.—Tr. arnica rad. . . . . . . . ℥ij.
Fl. ex. pilocarpin . . . . . ℥ij.
Spts. aeth. nit. . . . . . . . ℥iij.—M.

Sig.—Give one-half at a dose in a pint of water, and repeat in one hour.

I gave this mixture with the intention of dilating the cuticular bloodvessels, attracting the blood to the surface, and thus equalizing the circulation, following this up with a powerfully stimulating and diaphoretic mixture, the exact ingredients of which I do not recall. The mule recovered.

What ailed him? Was it a congestive chill; if so, what caused the vomiting? Was it a rupture of the mucous coat of the stomach; if so, how do you account for it without distention of that organ? I should add that he had been standing in the stable for a whole day, and the evidence of his not having been bloated is attested to by his owner and two hostlers.

CASE VI.—A fox-terrier dog got away from its owner for a short time (fifteen or twenty minutes), when he returned he seemed weak, and the next day blood was seen dripping from his penis. This condition persisted for a week, when I was called in.

Found the dog weak; mucous membrane pale; blood of arterial origin, coming from the urethral canal.

No history as to its cause; probably due to injury received during copulation, or possibly was run over by a bicycle.

Diagnosticated a rupture of the urethra and corpus spongiosum; for had the hemorrhage been due to injury of the kidneys or bladder, it would have been ejected only during urination; but in this case there was a constant dribbling. Prescribed

| | |
|---|---|
| ℞.—Ext. ergotæ fl. . . . . . . | ʒijj. |
| Ac. gallici . . . . . . . . | ʒss. |
| Ext. hamamel. fl. . . . . . . | ʒvj |
| Syr. aurantii cort. } Syr. glycyrrhizæ } . . . | āā qs. ad. ℥iij.—M. |

Sig.—Two teaspoonfuls every two or three hours.

*Locally.* Injection of a mild alum solution and bathing with cold water. The bleeding stopped in thirty-six hours, with no ill results.

